# Hypertonic saline and pentoxifylline enhance survival, reducing apoptosis and oxidative stress in a rat model of strangulated closed loop small bowel obstruction

**DOI:** 10.6061/clinics/2019/e787

**Published:** 2019-05-28

**Authors:** Gustavo Scapini, Roberto Rasslan, Natalie Chaves Cayuela, Miguel Angelo Goes, Marcia Kiyomi Koike, Edivaldo Massazo Utiyama, Edna Frasson de Souza Montero, Samir Rasslan

**Affiliations:** I Disciplina de Cirurgia Geral e Trauma, Departamento de Cirurgia, Faculdade de Medicina FMUSP, Universidade de Sao Paulo, Sao Paulo, SP, BR; II Divisao de Nefrologia, Universidade Federal de Sao Paulo (UNIFESP), Sao Paulo, SP, BR; III Disciplina de Emergencias Clinicas, Departamento de Clinica Medica, Faculdade de Medicina FMUSP, Universidade de Sao Paulo, Sao Paulo, SP, BR

**Keywords:** Apoptosis, Hypertonic Saline, Intestinal Obstruction, Oxidative Stress, Pentoxifylline

## Abstract

**OBJECTIVES:**

Intestinal obstruction has a high mortality rate when therapeutic treatment is delayed. Resuscitation in intestinal obstruction requires a large volume of fluid, and fluid combinations have been studied. Therefore, we evaluated the effects of hypertonic saline solution (HS) with pentoxifylline (PTX) on apoptosis, oxidative stress and survival rate.

**METHODS:**

Wistar rats were subjected to intestinal obstruction and ischemia through a closed loop ligation of the terminal ileum and its vessels. After 24 hours, the necrotic bowel segment was resected, and the animals were randomized into four groups according to the following resuscitation strategies: Ringer's lactate solution (RL) (RL-32 ml/kg); RL+PTX (25 mg/kg); HS+PTX (HS, 7.5%, 4 ml/kg), and no resuscitation (IO-intestinal obstruction and ischemia). Euthanasia was performed 3 hours after resuscitation to obtain kidney and intestine samples. A malondialdehyde (MDA) assay was performed to evaluate oxidative stress, and histochemical analyses (terminal deoxynucleotidyl transferase-mediated dUTP nick-end labeling [TUNEL], Bcl-2 and Bax) were conducted to evaluate kidney apoptosis. Survival was analyzed with another series of animals that were observed for 15 days.

**RESULTS:**

PTX in combination with RL or HS reduced the MDA levels (nmol/mg of protein), as follows: kidney IO=0.42; RL=0.49; RL+PTX=0.31; HS+PTX=0.34 (*p*<0.05); intestine: IO=0.42; RL=0.48; RL+PTX=0.29; HS+PTX=0.26 (*p*<0.05). The number of labeled cells for TUNEL and Bax was lower in the HS+PTX group than in the other groups (*p*<0.05). The Bax/Bcl-2 ratio was lower in the HS+PTX group than in the other groups (*p*<0.05). The survival rate on the 15^th^ day was higher in the HS+PTX group (77%) than in the RL+PTX group (11%).

**CONCLUSION:**

PTX in combination with HS enhanced survival and attenuated oxidative stress and apoptosis. However, when combined with RL, PTX did not reduce apoptosis or mortality.

## INTRODUCTION

Intestinal obstruction is a prevalent condition and accounts for 16% of surgeries in the emergency department. Morbidity and mortality are high in the presence of intestinal necrosis 1. Inflammation after intestinal cell death involves alterations in the absorption and secretion of liquids in the obstructed intestine and leads to a great loss of fluids. Increases in intraluminal content determine visceral distension and tissue perfusion impairment and are associated with altered permeability of the intestinal mucosa 2. This condition contributes to bacterial translocation, which is considered an important factor in the pathogenesis of sepsis 3-5.

Experimental models of intestinal obstruction have been studied to understand the physiopathology of systemic failure and provide advances in research to develop strategies for avoiding or attenuating this critical damage 3,4.

In intestinal obstruction, hypovolemia and sepsis can lead to acute kidney injury (AKI) and increased mortality. The main mechanisms that explain acute renal injury in sepsis include microvascular dysfunction, oxidative stress, inflammatory and immune responses, cellular hypoxia and apoptosis 6,7. The management of abdominal sepsis in complicated intestinal obstructions involves fluid resuscitation, early use of antibiotics and removal of ischemic small bowel 1. Stabilization of the hemodynamic framework requires the use of large amounts of fluid; however, the excessive administration of fluids generates extravasation into the interstitium and can have deleterious repercussions 8. Ringer's lactate (RL) is the most widely used crystalloid solution for volemic resuscitation, despite the fact that it can activate the inflammatory response 9-11.

Strategies to attenuate the inflammatory response during volume resuscitation have been studied and have mainly evaluated fluids used with or without added drugs. Hypertonic saline solution (HS) is attractive because it can modulate the immune and inflammatory response 12-15. Among the medications used with fluids, pentoxifylline (PTX) is a drug derived from methylxanthine that modifies the shape of erythrocytes, improves blood flow in the microcirculation, and decreases inflammation 16-18.

Coimbra et al. 19 studied the association of HS with PTX in toxin-stimulated blood cells and observed inhibition of neutrophil activation. Kim and Lee 20 previously reported the effects of the same treatment strategy in rats submitted to endotracheal injection of lipopolysaccharide and demonstrated lower production of proinflammatory cytokines. In our laboratory, in a rat model of intestinal obstruction, a reduction in NF-kB and inflammatory cytokines was observed after the administration of HS with PTX 21.

Although resuscitation in hemorrhagic shock and sepsis is highlighted in the literature, there are few studies that analyze the effects and consequences of resuscitation in complicated intestinal obstruction. Because bacterial translocation and hypovolemia are the main causes of organic dysfunction in intestinal obstruction, it was decided to evaluate the effects of HS with PTX on apoptosis, oxidative stress and survival in an experimental model of strangulated intestinal obstruction in rats.

## METHODS

The Ethics Committee of the Medical School of the University of São Paulo approved this study. The experiment was carried out at the Laboratory of Surgical Physiology (LIM-62) of the Medical School of the University of São Paulo. All animals were handled according to international protocols for the care, management and use of laboratory animals.

Sixty-four male Wistar rats weighing 250-300 g were purchased from the institutional animal house. Before and after the experiment, the animals were maintained in an environment controlled for temperature (23°C), humidity and exposure to artificial light (with a light-dark cycle of twelve hours) and had free access to food and water.

### Experimental protocol

Anesthesia was performed using ketamine (50 mg/kg) and xylazine (10 mg/kg), and then, the rats were submitted to a 3 cm median laparotomy. A rat model of intestinal obstruction was made in a closed loop by ligation of the terminal ileum at 10 cm and 1.5 cm from the ileocecal valve with 4-0 silk thread, and the vessels of this intestinal segment were occluded. Closure of the abdominal cavity was performed with 4-0 nylon sutures, as previously described 21.

After 24 hours of surgical trauma, the rats were submitted to a cervicotomy with cannulation of the jugular vein for volume resuscitation under anesthesia. Afterwards, the animals were randomized into four groups based on the resuscitation strategy. There were eight rats per group in each of the following groups: no resuscitation (IO-intestinal obstruction and ischemia); RL (32 ml/kg); RL (32 ml/kg) plus PTX (25 mg/kg); and HS (4 ml/kg) plus PTX (25 mg/kg). We used 32 ml/kg of Ringer's lactate solution (RL) and 4 ml of HS based on the Na^+^ concentration 21.

Immediately after resuscitation, the rats underwent a second laparotomy for resection of the ischemic intestinal segment, followed by anastomosis with 6-0 polypropylene sutures and then closure of the abdominal cavity with 4-0 nylon. Euthanasia was performed 3 hours after resuscitation, and samples of intestine and kidney were collected for MDA quantification. We performed Bax, Bcl-2 and terminal deoxynucleotidyl transferase-mediated dUTP nick-end labeling (TUNEL) analyses on the kidney samples.

Another series of 8 animals per group underwent surgery following the same protocols. These animals were used to evaluate survival after resuscitation and were euthanized at the 15^th^ postoperative day.

### Malondialdehyde assay

Intestinal and kidney tissues were processed to evaluate lipid peroxidation using the thiobarbituric acid reactive substances (TBARS) method, as previously described 22, expressed as nmol/mg protein.

### Apoptosis

We studied 4 μm thick renal tissue sections for the TUNEL, Bax and Bcl-2 assays. The slides were placed on silanized slides (Sigma Chemical Co., St. Louis, Missouri, USA) with suitable support. The dewaxing process was performed with warm xylol at a temperature of approximately 65°C for 10 minutes, and afterward, three cold xylol baths were used. The slides were washed in decreasing concentrations of absolute alcohol (95% and 70%) for hydration.

The slides were washed in water and deionized water and then preserved in pH 7.4 phosphate buffer (Synth®, São Paulo, Brazil). Proteinase K was used for 30 minutes for the recovery of the antigenic sites. Endogenous peroxidase blockade was made with 10% hydrogen peroxide in methanol for 30 minutes at room temperature and then washed with water, distilled water and pH 7.4 phosphate buffer.

The slides were incubated with 50 μl of the TUNEL assay reaction solution (5 μl of enzyme solution + 45 μl of buffer) per sample in a humid chamber at 37°C for 60 minutes. Afterward, the slides were washed in pH 7.4 phosphate buffer solution and incubated in a humidity chamber at 37°C for 30 minutes with 50 μl of the peroxidase converter. Subsequently, three baths in the buffer solution were performed. Staining was performed with diaminobenzidine, and counterstaining was performed with methyl green.

Recovery of the antigenic sites for evaluating Bax and Bcl-2 was performed using a solution of 10 mM citric acid and pH 6.0 in a humid bath under pressure at 125°C for one minute (Pascal®, DakoCytomation, Model: 2800, USA). The slides were incubated with the specific primary antibodies (anti-Bax and anti-Bcl-2) diluted in 1% bovine serum albumin for 24 hours at 4°C. Subsequently, they were incubated with the secondary antibody (Novolink Polymer, Leica Biosystems) and stained with diaminobenzidine (Sigma-Aldrich Chemie, Steinheim, Germany) and Harris hematoxylin (Merck®, Darmstadt, Germany).

The analysis of the slides prepared with the TUNEL, Bax and Bcl-2 reactions was performed blindly with optical microscopy, and 10 fields with a 40-fold increase were evaluated. The fields were photographed, and the labeled cells were counted as apoptotic events.

The Bax/Bcl-2 index was calculated by dividing the values obtained by counting in pairs, and it represents the relationship between the anti- and proapoptotic components present in the renal cells of each of the animals studied.

### Statistical analysis

Altman's nomogram was used to calculate the sample size, considering 80% as the level of statistical power and a *p*-value of 0.05.

Data are expressed as the median. Data analysis was performed with ANOVA on ranks and a post hoc test, when indicated (Student-Newman-Keuls test), using GraphPad Prism (GraphPad Software, San Diego, CA).

## RESULTS

### MDA levels

**Figure 1:**
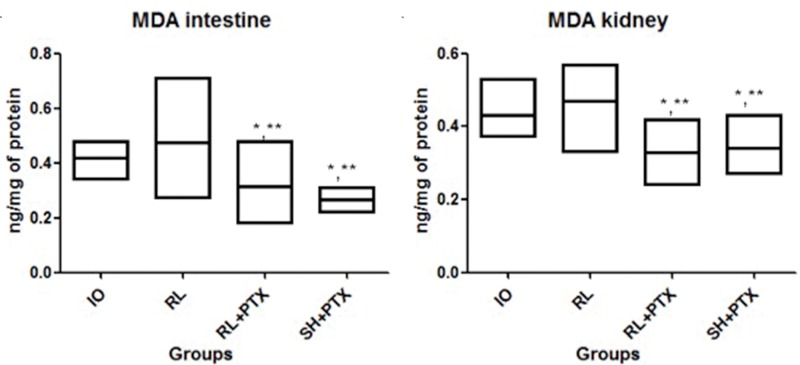
MDA concentrations (nmol/mg of protein) in the intestine (a) and kidney (b). Wistar rats were assigned to four groups: no resuscitation (IO-intestinal obstruction and ischemia), Ringer's lactate (RL), Ringer's lactate+pentoxifylline (RL+PTX), and hypertonic saline+pentoxifylline (HS+PTX). Data are expressed as the medians (interquartile range). (*p*<0.05) *versus IO; **versus RL; #*versus* RL+PTX.

Lipid peroxidation was attenuated by PTX when used with RL or HS (kidney: IO=0.42; RL=0.49; RL+PTX=0.31; HS+PTX=0.34; intestine: IO=0.42; RL=0.48; RL+PTX=0.29; HS+PTX=0.26; expressed as nmol/mg of protein). MDA levels in the RL+PTX and HS+PTX groups were similar in both organs. There was no difference between IO *vs*. RL in both organs (Figure 1).

### Apoptosis

**Figure 2:**
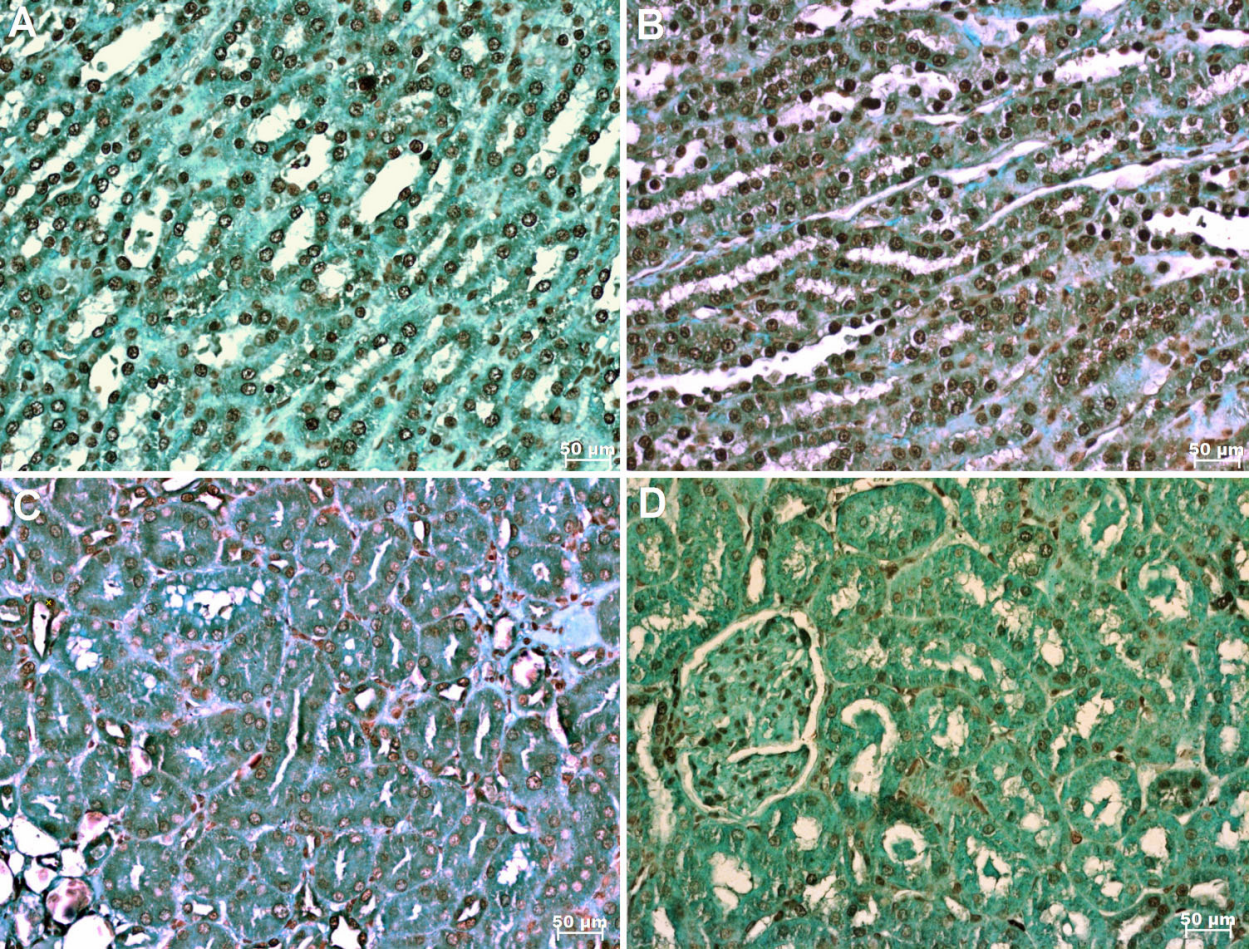
Photomicrography showing apoptosis in the kidney labeled by immunohistochemical staining (TUNEL; x 200). A. no resuscitation group (IO-intestinal obstruction and ischemia) B. Ringer's lactate group (RL) C. Ringer's lactate+pentoxifylline group (RL+PTX) D. Hypertonic saline+pentoxifylline group (HS+PTX).

**Figure 3:**
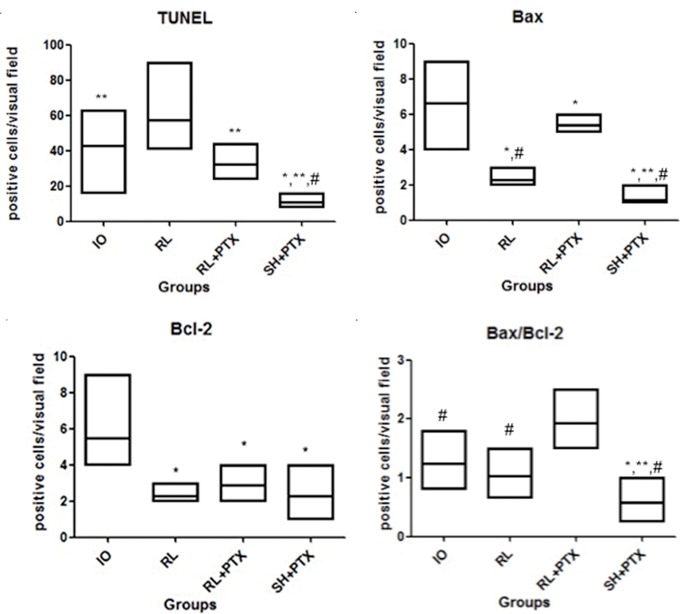
Expression of apoptosis in the kidney by immunohistochemical staining (positive cells/visual field): TUNEL (a), Bax (b), Bcl-2 (c), Bax/Bcl-2 (d). Wistar rats were assigned to four groups: no resuscitation (IO-intestinal obstruction and ischemia), Ringer's lactate (RL), Ringer's lactate + pentoxifylline (RL+PTX), and hypertonic saline+pentoxifylline (HS+PTX). The data are expressed as the medians (interquartile range). (*p*<0.05) *versus IO; **versus RL; #*versus* RL+PTX.

In the TUNEL assay, animals treated with HS+PTX showed decreased apoptosis compared to that in the other groups. In addition, PTX reduced apoptosis when used in combination with RL compared to when RL was used alone; moreover, compared with IO, RL alone was associated with increased apoptosis (Figure 2 and Figure 3a). Bax expression was also reduced by the combined use of HS+PTX compared with its expression in the other groups. However, Bax expression was increased when PTX was used in combination with RL. Compared to IO, the resuscitation strategy using RL was associated with decreased Bax levels (Figure 3b). Bcl-2, an antiapoptotic protein, demonstrated higher expression in IO than in any of the treated groups, which all showed similar expression of Bcl-2 (Figure 3c). The Bax/Bcl-2 ratio is illustrated in Figure 3d.

### Survival rates

**Figure 4:**
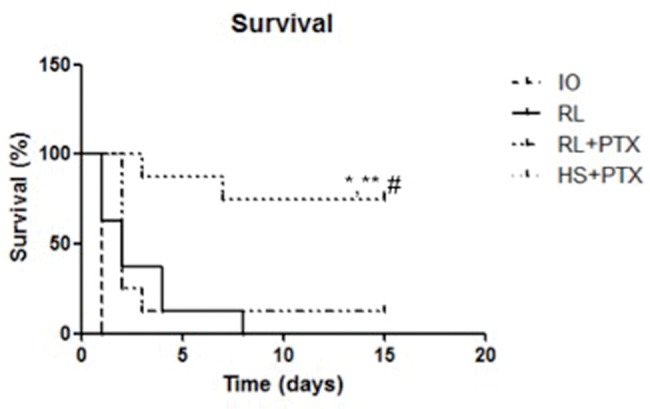
Kaplan-Meier survival analysis of animals. Wistar rats were assigned to four groups: no resuscitation (IO-intestinal obstruction and ischemia), Ringer's lactate (RL), Ringer's lactate+pentoxifylline (RL+PTX), and hypertonic saline+pentoxifylline (HS+PTX). Rats were observed for 15 days. (*p*<0.05) *versus IO; **versus RL; #*versus* RL+PTX.

Survival rates reached 77% in the HS+PTX group 15 days after the treatment. When used with RL, PTX did not affect survival compared with that of other groups (survival rate: up to 24 h—RL= 28%; RL+PTX= 31%; up to 15 days—RL=0%; RL+PTX=11%) (Figure 4).

## DISCUSSION

Resuscitation with HS and PTX caused a decrease in renal apoptosis and oxidative stress in the kidney and intestine. In addition, this therapeutic strategy resulted in increased survival during the treatment of abdominal sepsis. However, PTX with RL did not reverse the deleterious effects of RL.

Intestinal obstruction generates an intense inflammatory process that leads to an imbalance in the secretion of fluids, culminating in the loss of liquids and alterations in electrolytes 2. As assessed by other researchers, bacterial translocation is also a consequence of intestinal obstruction and may be the cause of sepsis in this condition 3,5,23. Moreover, intestinal involvement in sepsis involves the release of inflammatory factors that promote local and systemic lesions. Over the 24-hour period of intestinal obstruction and ischemia in this model, there was an increased inflammatory response and hemodynamic changes that implied a decrease in renal perfusion pressure 4.

In a model of intestinal obstruction in pigs, Fevang et al. 24 demonstrated that resuscitation with large volumes of fluids leads to an improvement in intestinal blood flow and less mucosal damage. However, it should be emphasized that excessive use of fluids in resuscitation predisposes patients to the occurrence of abdominal compartment syndrome and to a longer period of adynamic ileus 25,26. In the present study, the large volume of RL was bypassed by the use of HS in a volume eight times smaller.

Intestinal obstruction associated with ischemia causes damage that is aggravated by the correction of fluid volume. Damage to the mitochondrial membranes, endoplasmic reticulum or DNA triggers a complex process of production and release of proteins related to cell death, such as Bax (a proapoptotic protein) and Bcl-2 (an antiapoptotic protein). Bcl-2 combines with Bax or its binding sites in an attempt to disrupt the apoptotic cascade. When this balance is overcome by mechanisms of cell injury, particularly due to the formation of reactive oxygen and nitrogen species. Bax production increases, causing the release and activation of caspases and NF-kB and initiating the cell death process 27. Previous studies have shown that ischemia and reperfusion injury, hemorrhagic shock and sepsis are associated with increased cell death, either by necrosis or apoptosis 9,28,29.

Some studies have reported controversial results with regard to the occurrence of apoptosis after different resuscitation strategies using RL and HS. Deb et al. 9 demonstrated that volume resuscitation with RL in hemorrhagic shock leads to an increase in apoptosis, in contrast with SH, which does not alter the occurrence of apoptosis. In a hemorrhagic shock model in pigs, Rehberg et al. 30 observed a similar occurrence of apoptosis regardless of the type of solution used (RL or HS). In a burn model, Chen et al. 31 observed that HS reduced apoptosis and bacterial translocation. In the current study, the use of HS and PTX in combination (compared to RL alone) decreased the production of Bax, and based on the TUNEL analysis, this strategy downregulated apoptosis. It is interesting to observe that the IO group had higher values of the antiapoptotic protein Bcl-2, even surpassing the values of the HS+PTX group. However, Bax expression was also high and resulted in an increase in apoptosis. Therefore, the levels of Bax and Bcl-2 should be evaluated together, since the Bax/Bcl-2 ratio indicates the dynamics of apoptotic proteins 27. When analyzing this relationship in the kidney tissues, the HS with PTX group had a lower Bax/Bcl-2 ratio than that of the other groups.

Lipid peroxidation compromises the mechanisms of ionic exchange of the plasmatic membrane, leading to cell death 32. In the current study, there was increased lipid peroxidation in intestinal obstruction. The worsening of apoptosis with the use of RL as part of the resuscitation strategy was also accompanied by increased lipid peroxidation. In this model, in which the resuscitation and the surgery were delayed, the hypertonic solution used in combination with PTX led to a decrease in lipid peroxidation, apoptosis and mortality.

In a model of hemorrhagic shock with sepsis, it was observed that when PTX was administered with either RL or HS prior to cecal puncture, pulmonary inflammation was attenuated 16. Therefore, it was assumed that the same would occur in a model of intestinal obstruction and ischemia. However, PTX did not attenuate the proapoptotic effects of RL in this model. Perhaps this finding is due to the delayed treatment; in our model, we tried to reproduce what occurs in clinical practice when the patient arrives at the hospital with a certain time having already elapsed from the beginning of the insult. Corroborating the relevance of the timing of the therapeutic approach, Petroni et al. 13 found that the beneficial effects of HS in a sepsis model were related to early treatment. These authors demonstrated that when the resuscitation was delayed, both HS and RL increased mortality and inflammation.

We believe that it is essential to evaluate these strategies and their effects on oxidative stress and apoptosis with less aggressive insults and at earlier timepoints after treatment. The main limitation of our experimental protocol was that the 24-hour period after intestinal resection without resuscitation reflected a late phase of the injury and induced a 100% mortality rate. Depending on the timing of the intervention, the systemic inflammatory process that has already been established may not be reversed. Furthermore, the early analysis following treatment did not allow evaluation of the consequences of resuscitation. However, we evaluated the survival rates, which indirectly reflect the inflammatory response. Another criticism of the method is the absence of a group receiving HS without PTX. It may be questioned that the beneficial effects of the treatment are only due to the hypertonic solution. Actually, this question should be raised; however, our laboratory has already demonstrated that HS alone has positive effects in the treatment of complicated intestinal obstruction but that when associated with PTX, a synergistic effect is demonstrated 21. In the same way, this effect has already been shown by other authors in a model of hemorrhagic shock 33,34.

In conclusion, resuscitation with HS and PTX in an experimental model of small bowel obstruction with ischemia decreases lipid peroxidation, apoptosis and mortality. No benefits of PTX when used in combination with RL were observed in this experimental model.

## AUTHOR CONTRIBUTIONS

All authors involved in this study agree with the submission. Scapini G and Rasslan R were involved in the conception and design of the study, acquisition of the data, and analysis and interpretation of the data. Cayuela NC, Goes MA and Koike MK were involved in the acquisition and analysis of the data. Utiyama EM, Montero EF and Rasslan S were involved in manuscript drafting and critically review for important intellectual content.
